# Integrating Baduanjin exercise into Parkinson’s rehabilitation: a randomized controlled trial

**DOI:** 10.3389/fnagi.2026.1925159

**Published:** 2026-09-09

**Authors:** Muhammed Fatih Kavak, Nur Betül Civelek, Gülay Yalçın

**Affiliations:** 1 Department of Physiotherapy and Rehabilitation, Faculty of Health Sciences, Üsküdar University, İstanbul, Türkiye; 2 Institute of Health Sciences, Üsküdar University, İstanbul, Türkiye; 3 Department of Physiotherapy and Rehabilitation, Faculty of Health Sciences, Mudanya University, Bursa, Türkiye

**Keywords:** Baduanjin exercise, exercise therapy, Parkinson’s disease, physical therapy, postural balance, rehabilitation

## Abstract

**Objective:**

To evaluate whether adding Baduanjin exercise to conventional physiotherapy improves balance, functional mobility, walking capacity, and motor signs compared with conventional physiotherapy alone in nursing-home residents with Parkinson’s disease.

**Methods:**

This assessor-blinded, two-group randomized controlled trial included 30 participants allocated equally to conventional physiotherapy (CPT) or Baduanjin plus conventional physiotherapy (BDJ + CPT). CPT consisted of supervised 45-min sessions three times weekly for 12 weeks; the intervention group received an additional 15 min of supervised Baduanjin during each session. Twenty-seven participants completed follow-up and were included in the complete-case analysis (BDJ + CPT, *n* = 13; CPT, *n* = 14). Balance, functional mobility, walking capacity, and motor signs were assessed using the Berg Balance Scale (BBS), Timed Up and Go test (TUG), Six-Minute Walk Test (6MWT), and Movement Disorder Society-sponsored Unified Parkinson’s Disease Rating Scale Part III (MDS-UPDRS III), respectively. Because no single outcome had been designated *a priori* as primary, the four outcomes were analyzed in parallel using baseline-adjusted analysis of covariance (ANCOVA) with heteroskedasticity-consistent HC3 standard errors. Multiplicity was addressed using the Holm procedure.

**Results:**

At 12 weeks, BDJ + CPT was associated with a higher adjusted BBS score than CPT alone (adjusted mean difference, 9.93 points; 95% CI, 3.64 to 16.23; Holm-adjusted *p* = 0.006) and a greater 6MWT distance (38.76 m; 95% CI, 17.39 to 60.13; Holm-adjusted *p* = 0.002). The adjusted between-group difference in TUG favored BDJ + CPT but did not reach statistical significance (−4.73 s; 95% CI, −9.49 to 0.03; Holm-adjusted *p* = 0.103). No additional treatment effect was demonstrated for MDS-UPDRS III (1.78 points; 95% CI, −5.11 to 8.67; Holm-adjusted *p* = 0.613).

**Conclusion:**

In this small, single-site randomized trial, adding Baduanjin to conventional physiotherapy was associated with a higher BBS score and a longer 6MWT distance at 12 weeks after baseline adjustment. Additional benefits for TUG or MDS-UPDRS III were not demonstrated. Larger, prospectively powered trials are needed to determine the specific and clinically meaningful contribution of Baduanjin to Parkinson’s rehabilitation.

**Clinical trial registration:**

ClinicalTrials.gov, identifier NCT07306169.

## 1 Introduction

Parkinson’s disease (PD) is a progressive neurodegenerative disorder characterized by bradykinesia, rigidity, tremor, and postural instability, together with a broad range of non-motor manifestations. Impairments in balance, gait, transfers, and postural control contribute substantially to falls, activity limitation, and loss of independence, making exercise-based rehabilitation an important component of multidisciplinary management (Jankovic, 2008; Owen et al., 2019).

Physiotherapy interventions for PD commonly incorporate balance training, gait practice, strengthening, functional task training, and cueing strategies. Contemporary clinical practice guidance supports aerobic exercise, resistance training, balance training, gait training, and task-specific practice as components of physical therapist management in PD (Osborne et al., 2022). Although conventional exercise can improve several domains of physical function, no single exercise modality has consistently demonstrated superiority across all clinically relevant outcomes (Tomlinson et al., 2014).

Baduanjin is a traditional form of Qigong comprising a sequence of slow, coordinated movements involving controlled weight shifting, trunk and limb motion, breathing regulation, and sustained attentional engagement. Its low-impact nature allows movements to be adapted for older adults and people with reduced physical capacity. Previous randomized trials and systematic reviews have suggested potential benefits for balance, gait, mobility, and motor function in PD, although the magnitude of benefit has varied across outcomes and comparator conditions (Xiao and Zhuang, 2016; Chen et al., 2020; Lai et al., 2022).

Evidence from active-comparator studies is mixed. A fall-prevention study reported improvement in both Baduanjin and control groups but no significant between-group differences for several functional outcomes (Xiao et al., 2016). A meta-analysis found that benefits for motor symptoms and walking were less consistent against active than passive comparators (Chen et al., 2020). More recently, a 12-week randomized trial comparing Baduanjin with brisk walking found a between-group difference in MDS-UPDRS III but not BBS (Sun et al., 2026). These findings suggest that the incremental effects of Baduanjin may vary across functional domains and comparator conditions.

The present randomized controlled trial evaluated whether adding Baduanjin to conventional physiotherapy produced additional improvements in balance, functional mobility, walking capacity, and motor signs among nursing-home residents with PD. BBS, TUG, 6MWT, and MDS-UPDRS III were evaluated as parallel clinical outcomes.

## 2 Materials and methods

### 2.1 Study design and setting

This prospective, assessor-blinded, two-group parallel randomized controlled trial compared conventional physiotherapy (CPT) with Baduanjin exercise added to conventional physiotherapy (BDJ + CPT) in older nursing-home residents with PD. The study was conducted at Kadim Nursing Home Elderly Care Center between January and June 2024. Assessments were performed before the intervention (T0) and after the 12-week intervention period (T1). Outcome assessments were performed by an independent physiotherapist who was not involved in intervention delivery and remained blinded to group allocation. Exercise sessions were delivered by a separate physiotherapist.

The study was approved by the Üsküdar University Non-Interventional Clinical Research Ethics Committee on 29 December 2023 (approval no. 61351342/Aralık 2023-12). Written informed consent was obtained before enrollment.

### 2.2 Participants and eligibility

Nursing-home residents aged 65 years or older with a clinical diagnosis of PD were considered for participation. Participants were required to provide informed consent and demonstrate sufficient ability to understand and safely perform exercise instructions. This ability was evaluated at baseline by a trained physiotherapist using a structured clinical assessment applied consistently to all participants. The assessment required participants to understand and execute multistep exercise instructions and to participate safely in planned activities. No standardized cognitive screening instrument such as the Mini-Mental State Examination or Montreal Cognitive Assessment was administered.

Exclusion criteria were unstable cardiovascular disease; visual or hearing impairment severe enough to prevent adequate communication or exercise instruction; severe osteoarthritis, recent fracture, or another marked musculoskeletal limitation preventing safe participation; inability to understand or follow instructions in Turkish; or inability or unwillingness to continue participation.

### 2.3 Randomization, allocation concealment, and masking

Participants were allocated in a 1:1 ratio using a computer-generated blocked randomization sequence. The allocation list was held by an independent researcher, and investigators responsible for enrollment and baseline assessment did not have access to the list before treatment assignment. A sealed-envelope system was not used. The outcome assessor remained blinded to group allocation. The treating physiotherapist was necessarily aware of the assigned exercise program.

### 2.4 Sample size and outcome hierarchy

The study protocol specified a target enrollment of 30 participants. No formal *a priori* sample-size calculation was documented. No single outcome measure was designated *a priori* as the primary outcome. BBS, TUG, 6MWT, and MDS-UPDRS III were analyzed as parallel clinical outcomes, and family-wise multiplicity across the four outcomes was controlled using the Holm procedure.

### 2.5 Intervention programs

Participants assigned to CPT received 45-min supervised physiotherapy sessions three times per week for 12 weeks. Each session comprised 5 min of slow walking as warm-up, 15 min of balance-oriented activities, 10 min of strengthening exercises, 10 min of gait training, and 5 min of breathing exercises. Functional training included level walking, stair negotiation, sit-to-stand transfers, backward and toe walking, single-leg stance, tandem and lateral walking, turning, and supported weight shifting. Balance activities incorporated balls and resistance bands when appropriate. Strengthening included squatting, lower-limb cycling-type movements, and resistance exercises adapted to individual physical ability. Gait practice emphasized step length and directional changes. These components are consistent with contemporary physical therapy recommendations for PD (Osborne et al., 2022).

Participants assigned to BDJ + CPT received the same 45-min CPT program plus an additional 15-min supervised Baduanjin session three times per week for 12 weeks. The adapted Baduanjin program incorporated controlled breathing, coordinated upper- and lower-body movements, trunk opening and lateral stretching, trunk rotation, lateral flexion, controlled knee flexion with reaching toward the feet, shoulder lifting and relaxation, and coordinated arm circles. Movements were generally repeated 6–8 times according to participant tolerance. Similar Baduanjin programs have been evaluated in PD populations (Xiao and Zhuang, 2016; Lai et al., 2022). No formal session-level adherence metric or prespecified quantitative progression algorithm was recorded (see Table 1).

**Table 1:** Intervention content and scheduled dose.

Component	CPT	BDJ + CPT	Scheduled dose	Adaptation / progression	Supporting reference
Slow-walking warm-up	Yes	Yes	5 min/session	According to tolerance	Osborne et al. (2022)
Balance activities	Yes	Yes	15 min/session	Adapted to functional ability	Osborne et al. (2022)
Strengthening	Yes	Yes	10 min/session	Resistance adapted individually	Osborne et al. (2022)
Gait training	Yes	Yes	10 min/session	Step length and directional changes	Osborne et al. (2022)
Breathing exercises	Yes	Yes	5 min/session	According to tolerance	Study intervention schedule
Baduanjin	No	Yes	15 min/session	6–8 repetitions according to tolerance; no formal progression algorithm recorded	Xiao and Zhuang (2016) and Lai et al. (2022)
Total scheduled session time	45 min	60 min	3 sessions/week for 12 weeks	Supervised	Study intervention schedule

### 2.6 Outcome measures

Functional balance was assessed with the 14-item Berg Balance Scale (BBS), in which each item is scored from 0 to 4 and total scores range from 0 to 56, with higher scores indicating better balance performance (Berg et al., 1992).

Functional mobility was assessed using the Timed Up and Go test (TUG). Participants rose from a chair, walked 3 m, turned, returned to the chair, and sat down; time was recorded in seconds, with shorter times indicating better mobility (Podsiadlo and Richardson, 1991).

Walking capacity was assessed using the Six-Minute Walk Test (6MWT), with total distance walked in 6 min recorded in meters (Guyatt et al., 1985).

Motor signs were evaluated using Part III (Motor Examination) of the Movement Disorder Society-sponsored Unified Parkinson’s Disease Rating Scale (MDS-UPDRS III), with higher scores indicating greater motor impairment (Goetz et al., 2008).

### 2.7 Statistical analysis

Continuous outcomes are presented as mean ± standard deviation. The inferential analyses focused on between-group effects at 12 weeks. For each outcome, an analysis of covariance (ANCOVA) model was fitted with the 12-week outcome as the dependent variable, group assignment as the main independent variable, and the corresponding baseline outcome as a covariate. The group coefficient represents the baseline-adjusted mean difference between BDJ + CPT and CPT at 12 weeks.

The homogeneity-of-regression-slopes assumption was assessed using a group-by-baseline interaction term. Given the small sample and evidence of heteroscedasticity in some models, heteroskedasticity-consistent HC3 standard errors were used for inference. Adjusted mean differences are reported with 95% confidence intervals. The Holm procedure was used to control the family-wise error rate across the four parallel outcomes; both unadjusted and Holm-adjusted two-sided *p* values are reported. No imputation was performed for participants without 12-week data; a complete-case approach was used. Analyses were conducted using Python 3.13.5 and statsmodels 0.14.6. Generative AI assistance used for analysis-code generation is described in the Generative AI statement.

## 3 Results

### 3.1 Participant flow

**Figure 1:**
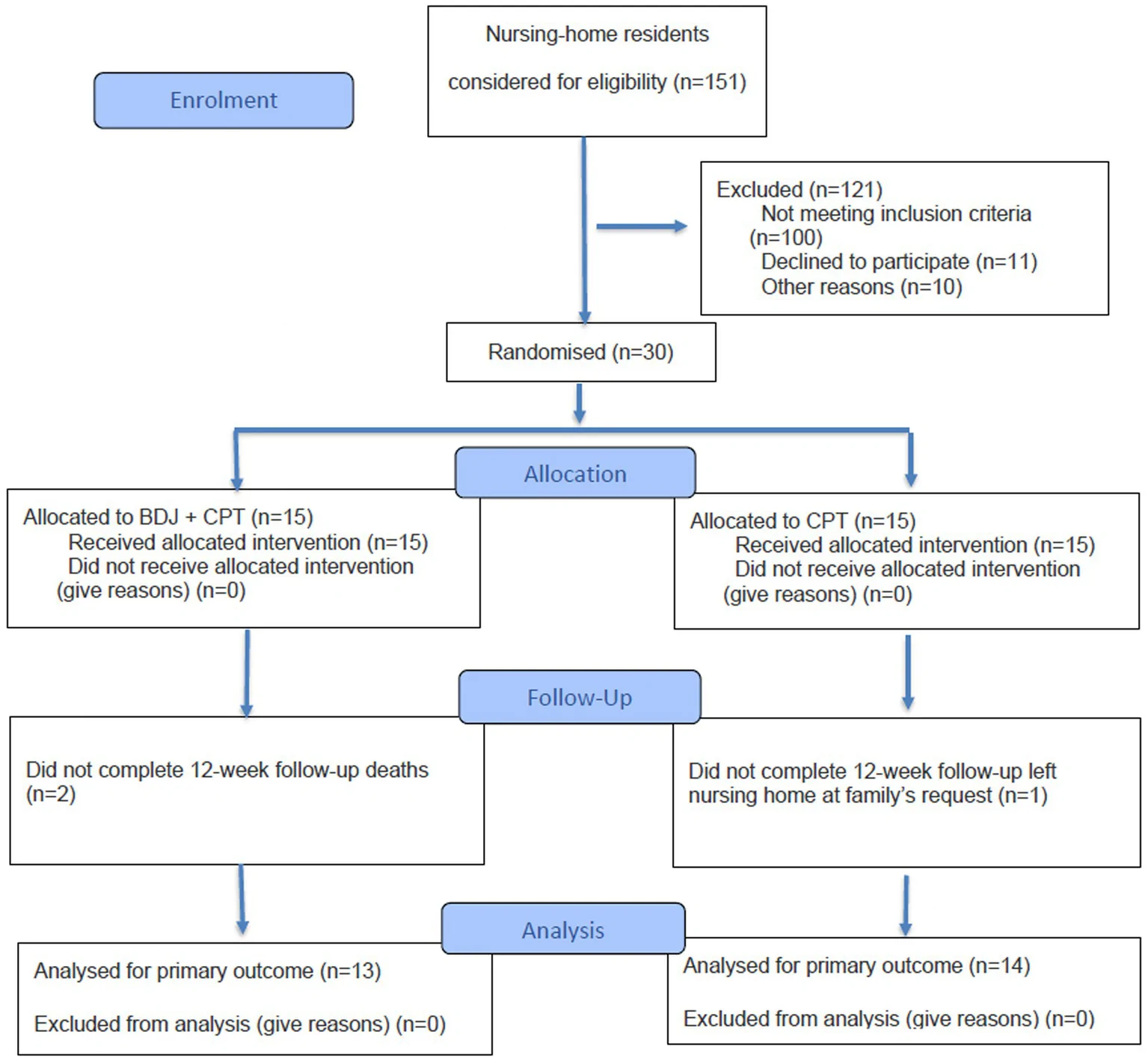
Participant flow diagram. BDJ + CPT, Baduanjin plus conventional physiotherapy; CPT, conventional physiotherapy.

Of 151 nursing-home residents considered for eligibility, 30 participants were randomized in a 1:1 ratio to BDJ + CPT (*n* = 15) or CPT (*n* = 15). Individual reasons for non-randomization among the remaining 121 residents were not available in the study records. During follow-up, two participants in the BDJ + CPT group died and one participant in the CPT group left the nursing home at the family’s request. Complete 12-week outcome data were therefore available for 27 participants: 13 in BDJ + CPT and 14 in CPT. Participant flow is shown in Figure 1.

### 3.2 Baseline clinical outcomes

At baseline, mean BBS scores were 12.69 ± 12.27 in BDJ + CPT and 13.71 ± 12.26 in CPT. Mean TUG times were 24.62 ± 10.67 s and 32.64 ± 13.76 s, respectively; mean 6MWT distances were 53.62 ± 21.69 m and 54.21 ± 20.02 m; and mean MDS-UPDRS III scores were 51.62 ± 27.18 and 68.14 ± 38.26. Baseline outcome values are summarized in Table 2.

**Table 2:** Baseline clinical outcome measures among participants included in the complete-case analysis.

Outcome	BDJ + CPT (*n* = 13)	CPT (*n* = 14)
BBS, points	12.69 ± 12.27	13.71 ± 12.26
TUG, s	24.62 ± 10.67	32.64 ± 13.76
6MWT, m	53.62 ± 21.69	54.21 ± 20.02
MDS-UPDRS III, points	51.62 ± 27.18	68.14 ± 38.26

### 3.3 Baseline-adjusted treatment effects

For BBS, the baseline-adjusted mean difference at 12 weeks favored BDJ + CPT by 9.93 points (95% CI, 3.64 to 16.23; *p* = 0.002; Holm-adjusted *p* = 0.006). Mean BBS scores were 12.69 ± 12.27 at baseline and 36.08 ± 13.57 at week 12 in BDJ + CPT, compared with 13.71 ± 12.26 and 26.86 ± 9.10 in CPT.

For 6MWT, the baseline-adjusted mean difference at 12 weeks favored BDJ + CPT by 38.76 m (95% CI, 17.39 to 60.13; *p* < 0.001; Holm-adjusted *p* = 0.002). Mean 6MWT distances were 53.62 ± 21.69 m at baseline and 100.85 ± 43.59 m at week 12 in BDJ + CPT, compared with 54.21 ± 20.02 m and 62.79 ± 25.33 m in CPT.

For TUG, the baseline-adjusted mean difference was −4.73 s (95% CI, −9.49 to 0.03; *p* = 0.052; Holm-adjusted *p* = 0.103) and did not reach statistical significance. Mean TUG times were 24.62 ± 10.67 s at baseline and 15.08 ± 6.95 s at week 12 in BDJ + CPT, compared with 32.64 ± 13.76 s and 24.00 ± 9.10 s in CPT.

For MDS-UPDRS III, the baseline-adjusted mean difference was 1.78 points (95% CI, −5.11 to 8.67; *p* = 0.613; Holm-adjusted *p* = 0.613), providing no evidence of an additional between-group benefit. Mean MDS-UPDRS III scores were 51.62 ± 27.18 at baseline and 32.62 ± 20.81 at week 12 in BDJ + CPT, compared with 68.14 ± 38.26 and 44.79 ± 35.52 in CPT.

Treatment-by-baseline interaction terms were not statistically significant for any outcome (all *p* values ≥ 0.197) (see Table 3).

**Table 3:** Baseline-adjusted treatment effects at 12 weeks.

Outcome	BDJ + CPT baseline	BDJ + CPT week 12	CPT baseline	CPT week 12	Adjusted mean difference (95% CI)	*p* value	Holm-adjusted p value
BBS, points	12.69 ± 12.27	36.08 ± 13.57	13.71 ± 12.26	26.86 ± 9.10	+9.93 (3.64 to 16.23)	0.002	0.006
TUG, s	24.62 ± 10.67	15.08 ± 6.95	32.64 ± 13.76	24.00 ± 9.10	−4.73 (−9.49 to 0.03)	0.052	0.103
6MWT, m	53.62 ± 21.69	100.85 ± 43.59	54.21 ± 20.02	62.79 ± 25.33	+38.76 (17.39 to 60.13)	<0.001	0.002
MDS-UPDRS III, points	51.62 ± 27.18	32.62 ± 20.81	68.14 ± 38.26	44.79 ± 35.52	+1.78 (−5.11 to 8.67)	0.613	0.613

Adjusted mean differences are BDJ + CPT minus CPT from ANCOVA models adjusted for the corresponding baseline value using HC3 robust standard errors. Positive values favor BDJ + CPT for BBS and 6MWT; negative values favor BDJ + CPT for TUG. Lower MDS-UPDRS III scores indicate better motor function.

## 4 Discussion

Adding 15 min of supervised Baduanjin to conventional physiotherapy was associated with better 12-week balance and walking-capacity outcomes after adjustment for baseline values and multiplicity. Between-group differences were evident for BBS and 6MWT, whereas TUG and MDS-UPDRS III did not show statistically significant additional effects. The findings therefore suggest an outcome-specific rather than uniform incremental effect.

The adjusted BBS difference was approximately 10 points in favor of BDJ + CPT. This exceeds the 5-point minimal detectable change (MDC95) reported for BBS in people with parkinsonism (Steffen and Seney, 2008). However, MDC reflects change beyond measurement error at the individual level and is not equivalent to a group-level minimal clinically important difference. The BBS finding should therefore be interpreted as supporting a potentially meaningful balance effect rather than as definitive evidence of clinical benefit.

The adjusted 6MWT difference was approximately 39 m. Steffen and Seney (2008) reported an MDC95 of 82 m for 6MWT in people with parkinsonism, which is larger than the present between-group estimate. This does not negate the statistically significant between-group difference, but it indicates that clinical importance cannot be inferred from statistical significance alone and that measurement thresholds derived from other PD populations may not transfer directly to institutionalized older adults.

The TUG point estimate favored BDJ + CPT by approximately 4.7 s, numerically exceeding the 3.5-s MDC reported in PD (Huang et al., 2011). Nevertheless, the 95% confidence interval included essentially no between-group difference, and neither the unadjusted nor Holm-adjusted result reached statistical significance. The trial therefore does not provide sufficiently precise evidence of an incremental TUG benefit.

No additional improvement was demonstrated for MDS-UPDRS III. Horváth et al. (2015) estimated a minimal clinically important improvement of approximately 3.25 points for MDS-UPDRS III. The present adjusted treatment estimate was 1.78 points in the direction of a higher, rather than lower, score in BDJ + CPT and had a confidence interval spanning potential benefit and harm. The result therefore does not support an incremental effect on global motor signs.

These results partly align with earlier Baduanjin research but also illustrate inconsistency in the literature. Xiao and Zhuang (2016) reported improvements in BBS, 6MWT, and TUG following a six-month Baduanjin program. A systematic review and meta-analysis also reported pooled improvements in MDS-UPDRS III, BBS, and 6MWT, but the included trials varied in comparator type, intervention dose, disease severity, and outcome protocols (Lai et al., 2022). Shorter-duration research has likewise suggested potential effects on selected domains such as fine motor performance and quality of life (Li et al., 2024).

Evidence from active-control comparisons is less uniform. Xiao et al. (2016) reported improvements over time but no between-group differences for several functional measures in a fall-prevention study. Chen et al. (2020) found that Qigong benefits for motor symptoms and walking were significant against passive controls but not active controls, whereas balance effects were more consistent. In a recent randomized trial comparing Baduanjin with brisk walking, Baduanjin produced greater improvement in MDS-UPDRS III but not BBS (Sun et al., 2026). Together, these studies suggest that the incremental effects of Baduanjin may depend on outcome domain and comparator intensity.

Because both groups received active conventional physiotherapy, the between-group contrast estimates the incremental effect of adding Baduanjin rather than a comparison with no structured exercise. BDJ + CPT nevertheless included 15 additional minutes of supervised exercise per session. The observed differences therefore cannot be attributed with certainty to Baduanjin-specific movement characteristics rather than to greater total supervised exercise exposure.

The nursing-home setting is another important contextual feature. Institutionalized individuals may differ from community-dwelling people with PD in baseline mobility, frailty, environmental support, and rehabilitation exposure. The comparatively low baseline BBS and 6MWT values suggest that the functional profile of this sample may not resemble that of many community-based exercise cohorts. The findings should therefore be viewed as preliminary evidence for selected physical-function outcomes in institutional rehabilitation rather than as evidence of broad superiority across PD motor domains.

## 5 Limitations

This study has several limitations. First, the sample was small, no formal *a priori* sample-size calculation was documented, and no single clinical outcome had been designated prospectively as primary. Four outcomes were therefore evaluated in parallel with Holm adjustment for multiplicity, but confidence intervals remained wide for TUG and MDS-UPDRS III. Second, analyses were based on 27 participants with complete baseline and 12-week data rather than a strict intention-to-treat population. Three randomized participants were not included in the final analysis, including two deaths in BDJ + CPT and one participant in CPT who left the nursing home, introducing potential attrition bias. Because adverse events were not collected using a systematic surveillance protocol and cause/relatedness information was not available for safety adjudication, the two deaths cannot be interpreted as related or unrelated to the intervention. Third, all participants were recruited from a single nursing home, limiting external validity for community-dwelling populations. Fourth, BDJ + CPT included 15 min more supervised exercise per session than CPT, so the study cannot distinguish Baduanjin-specific effects from additional exercise time or therapist contact. Fifth, formal session-level adherence data and prespecified progression metrics were not recorded, preventing quantitative assessment of intervention fidelity. Sixth, individual Hoehn and Yahr stage data were unavailable, preventing reliable disease-stage counts or severity subgroup analyses. Seventh, cognitive eligibility was based on a structured clinical assessment rather than a standardized cognitive screening instrument. Eighth, the trial was registered retrospectively after study completion; the registry therefore does not provide prospective documentation of outcome hierarchy. Finally, follow-up was limited to 12 weeks, and persistence of effects, falls, independence, and quality of life were not evaluated.

## 6 Conclusion

In this small randomized trial of nursing-home residents with Parkinson’s disease, adding Baduanjin to conventional physiotherapy was associated with a higher baseline-adjusted BBS score and a longer 6MWT distance at 12 weeks. Additional benefits were not demonstrated for TUG or MDS-UPDRS III. The small sample, complete-case analysis, unequal supervised exercise time, absence of systematic adverse-event monitoring, and lack of long-term follow-up limit the strength and generalizability of these findings. Larger, prospectively powered trials with equal-duration active comparators are needed to determine the specific and clinically meaningful contribution of Baduanjin to Parkinson’s rehabilitation.

## Data Availability

The raw data supporting the conclusions of this article will be made available by the authors, without undue reservation.
